# Life Insurance

**Published:** 1867-08

**Authors:** W. W. Jones

**Affiliations:** Toledo, Ohio


### Correspondence.

### Life Insurance.

BY W. W. JONES, M. D., TOLEDO, OHIO.

Your article on "Examinations for Life Insurance," in the July number, suggests some further thoughts to which the attention of the profession may properly be called.

In theory, at least, the medical examiner holds his appointment direct from the home office of the company, and is supposed to hold an independent position, and be the guardian of its interest, and it would be considered undignified in him to fail to make an honest report of the cases he examines. Under the present plan or rules adopted by insurance companies, the whole of the examiner's report is subject to the inspection and criticism of the agent and applicant, both of whom are anxious that it should be most favorable. In case an unfavorable or doubtful report is necessarily made, the agent notices it, and the question naturally arises, how can this applicant be got through? If the company has more than one examiner, (and in some instances they have as many as the agent chooses to name,) the applicant performs a pilgrimage. The doctor who unequivocally recommends him for a policy is the best fellow in the estimation of both the applicant and the agent, and gradually gets the most of the examinations to do. An agent of one of the most prominent companies recently informed me that it was absolutely neeessary to have some *Dutch examiners because the competition was so great;* this company already had several other examiners, not all of whom would be recognized by the profession as medical. I knew a general agent to instruct his solicitors "that when they found that the medical examiner did not work for the *interest of the agent*, to get one who would;" and by reading the report made by the physician they are enabled to judge well how the application will be decided at the home office. They never think it worth while to send on any application that will probably be rejected; neither do they wish to pay the fee.

So long as the interest of the medical examiner and agent are diverse, that of the former depending upon his duty to those he represents, and that of the latter upon premiums, and these upon the acceptance of the application, these things will happen, unless radical changes are made in the present mode of doing business.

The interest of the profession as well as the companies they represent, demands that the present system of medical examinations be more completely divorced from the agencies, and that the examiners be made entirely independent of the agents. The medical examiner should report his examination direct to the home office, and not permit the agent or applicant to inspect his report. Much that it may contain may be of such a nature (especially if unfavorable to the applicant,) as to create ill feeling, repeated examples of which have happened it is believod to all, who have had much experience in making examinations for life insurance. Advantage is often taken to the prejudice of the medical examiner from the heedless disclosure of facts and opinions which his duty makes it necessary to point out in the answers and results of his examination. I believe, that the experience of most physicians who have been examiners will admit the assertion that their losses from these causes, will go far towards balancing their receipts from the fees for such examinations.

Let it not be inferred from the foregoing that I intend to charge agents with dishonest or reprehensible practices. So long as life insurance companies permit the present system to exist they only take advantage of what they consider as legitimate means to do the most business, and while some resort to dishonest means to get a risk accepted, they are the exception and not the rule.

The certificate of the family physician of the party as commonly obtained, is valueless. The company do not pay him, neither do they keep his counsels. If he has any knowledge which he has obtained through his professional intercourse with his patient that would operate against his obtaining an insurance, it would be against his interest to divulge it, and especially if it were put in pen and ink and subjected to the criticism of an agent. I recollect the first instance where I filled out such a certificate, which was rejected on my statements of facts; it cost me the loss of practice in a family worth over a hundred dollars a year, and I did not even get the fee for the certificate.

The medical examiner would be best able to judge after examining the applicant whether the family physician's statement would be necessary, and if so, he should be authorized to obtain and pay for it, and treat it as confidential.

I cordially endorse the views you take in relation to the duties of life insurance companies to the profession, and that their permanence depends upon surrounding themselves with all the safeguards—or in other words, seeing to it, *that they take only the best risks.*

